# Supplementary material for: Are carbon emissions trading and green financial instruments synergistic? -Comprehensive quantitative research based on content analysis
Source: PLoS One. 2024 Mar 7;19(3):e0298601. doi: 10.1371/journal.pone.0298601 (PMC10919601; doi:10.1371/journal.pone.0298601)
Supplement: S1 Data — (ZIP) [file pone.0298601.s001.zip › Data/地级市Z_F工作报告.zip!/╔╜╢1⁄2/║╩╘≤╩╨/└┤╫╘╣1⁄2╓┌║┼í╛┬φ┐╦╩2╛▌═°í┐_2015.docx]

　　一、2014年工作回顾

　　过去的一年，全市上下在中共菏泽市委的坚强领导下，认真贯彻中央和省委、省政府的决策部署，以提高质量效益为中心，以开展党的群众路线教育实践活动为动力，牢牢把握发展大势，全面推进各项改革，扎实推动工作落实，较好地完成了经济社会发展目标。

　　——经济平稳较快发展。实现地区生产总值2222.2亿元，增长10.2%。三次产业比调整为11.9:53.6:34.5，第三产业占比提高1.3个百分点。完成公共财政预算收入162亿元，增长1.7%，税收和主体税种占比分别达到76.5%、58.8%。城镇居民人均可支配收入达到23344元，增长9.9%；农民人均纯收入达到10436元，增长12.1%。进出口总额完成35.2亿美元，增长19%。金融机构存、贷款余额分别比年初增加309.4亿元、216.3亿元，新增社会信用总量373亿元。现代农业持续发展。粮食总产140.45亿斤，实现“十一连增”。规模以上农业龙头企业达到1426家，农业标准化基地新增50万亩，农民合作社、家庭农场分别新增8780家、322家，农作物耕种收综合机械化水平提高2个百分点。新增千亩以上牡丹种植大户22家，深加工企业发展到120家。工业经济平稳增长。规模以上工业主营业务收入、利税分别增长15.4%和11.6%。主营业务收入过50亿元、入库税金过亿元的企业分别达到11家、10家。四大工业主导产业主营业务收入、利税分别增长15.7%和12.2%。服务业繁荣发展。服务业增加值增长11.7%，社会消费品零售总额增长13%，商贸物流业交易额增长14%，旅游总收入增长16.8%。

　　——发展后劲持续增强。招商引资成效显著。引进落地亿元以上项目403个，完成投资710亿元，竣工投产230个；新批外资企业13家，实际到位外资2.2亿美元。项目建设扎实推进。省重点项目个数居全省第二位，100个市重点项目顺利实施，60个竣工投产；规模以上固定资产投资完成940.7亿元，增长16%。市场主体快速增加。新登记个体工商户6.4万户、私营企业9809家，分别增长144%和117%，规模以上工业企业增加227家。科技创新步伐加快。研发投入占地区生产总值的比重提高到1.1%，高新技术产业产值占比接近全省平均水平，省级以上科技创新平台达到81家，在建科技企业孵化器7家。重大基础设施建设实现新突破。菏泽火车站及广场改造提前完工，洙水河航道实现贯通，洙赵新河治理全面完成，菏泽机场选址报告通过国家民航总局评审，菏泽至曲阜城际铁路项目建议书获批，青岛保税港区菏泽功能区正式签约，济南铁路局菏泽铁路物流园区启动建设，巨野宝源湖水库、东明洪源水库建成蓄水，华润东明热电联产项目被列入省火电建设规划。

　　——城乡建设步伐加快。城镇化率达到43.1%，提高1.5个百分点。8个县城、首批6个省级示范镇控制性规划和第二批11个省级示范镇总体规划全部完成，农村新型社区规划实现全覆盖。市区实施城建重点工程40项，打通断头路5条，长城路西起昆明路东至广州路、西安路南起长江路北至北外环全程贯通，丹阳立交桥东段引桥基本完工，埋设雨污管网50公里，新增集中供热面积400万平方米。4个县完成农村公路网格化工程，新建改造农村公路2195公里。新建农村新型社区52个。省级示范镇和市级重点镇建成区面积增加12.9平方公里。新增农村污水处理站126个、垃圾中转站159座，村收集、镇转运、县区处理的垃圾处理体制初步形成。

　　——生态建设成效显著。大力实施绿色、循环、低碳发展战略，拒批“两高”项目22个，实施节能技改项目96个，淘汰高耗能落后设备1100台套，累计节能37万吨标煤。挖潜盘活存量建设用地1.23万亩，新增耕地1.06万亩，完成造林面积28.2万亩。创新实施生态补偿和环境污染有奖举报办法，积极推行大气污染防治网格化管理，严格落实“河长制”，生态环境持续改善。三条主要出境河流断面水质稳定达标；“蓝天白云”天数在全省前移7个位次，增加天数全省第一。

　　——各项改革扎实推进。继续深化行政审批、注册登记、财税金融、教育卫生等领域改革，启动改革事项367个。编制完成了市级行政审批事项目录，取消行政事业性收费项目100个，审批事项和时限进一步压减。农村产权制度改革迈出新步伐，土地承包经营权确权登记颁证完成84.3%。义务教育综合改革扎实推进，牡丹区、成武县农信社改制为农商行，“四位一体”公共资源市场化配置改革成效明显，城乡医疗保险整合、养老体制并轨、户籍制度改革、县级公立医院改革走在全省前列，郓城县、牡丹区分别被列为国家新型城镇化综合试点、全省县域经济科学发展试点。

　　——民生保障水平日益提高。民生支出占公共财政预算支出的比重继续提高，达到76.9%。年初确定的30件民生实事，除2件调整计划外，均已完成。全市新增安全饮水人口100万人，新增城镇就业9.5万人、农村劳动力转移就业24万人。城乡低保人均年保障标准分别提高120元、400元，五保供养和大病救助标准分别提高500元、8000元，每千名老人拥有社会养老床位达到26张。完成财政性教育投入65.5亿元，新建改建农村义务教育学校校舍121万平方米，新增幼儿园227所、城区中小学14所，齐鲁工业大学菏泽校区实现首批招生。市区新上新能源公交车100辆、公共自行车2000辆。县区“两馆”全部竣工，新建农村文化大院356个、文化广场329个、村级健身工程1055处、乡镇健身广场50处。开工建设保障性住房1.54万套，改造农村危房9032户。新增医疗床位3200张，市立医院、单县中心医院被评为“三甲”综合医院，计划生育单独两孩政策稳妥实施。精准扶贫全面铺开，31.2万人实现脱贫。加强和创新社会治理，积极排查化解矛盾纠纷，强化社会治安综合治理，严格安全生产，加强食品药品监管，全市没有发生恶性刑事案件、大规模群体性事件和重大安全事故，人民群众的安全感、满意度进一步提升。

　　——政府自身建设不断加强。扎实开展群众路线教育实践活动，着力查改“四风”问题，强化“庸懒散”治理和督查问效，工作效率、服务水平明显提升。实行市长公开电话24小时接听，开展行风热线和网络问政，拓展了社情民意反映渠道。建立了政府法律顾问制度，密切与各民主党派、工商联、无党派人士对口联系，决策水平进一步提高。自觉接受人大的法律监督、工作监督和政协的民主监督，人大代表建议和政协提案办复率100%。严格落实党风廉政建设责任制，认真贯彻中央八项规定精神，严查公款吃喝、公车私用，“三公”经费大幅下降，机关风气显著好转。

　　妇女儿童、关心下一代、残疾人、老龄、红十字会、慈善等事业持续发展，民族宗教、外事侨务、人民武装、防震减灾、邮政通讯、气象、人防、统计、物价、对台、史志、档案等工作都取得新的成绩。

　　成绩来之不易，成绩凝聚着全市人民的心血和汗水。在此，谨代表市人民政府，向勤劳智慧的全市人民，向各级人大代表、政协委员，向各民主党派、工商联、人民团体和社会各界人士，向离退休老领导、老同志，向驻菏部队、武警官兵、公安干警，向中央、省驻菏单位，向所有关心支持菏泽发展的同志们、朋友们，表示衷心的感谢并致以崇高的敬意！

　　各位代表，我市经济社会发展中还存在一些困难和问题：生产要素制约加剧，投资动力不足，招商引资、项目建设难度加大。重化工比重高，创新能力弱，产品大多处于价值链低端，市场竞争力不强。土地利用水平低，节能减排任务重，生态环境质量与群众期盼相比还有差距。社会治理存在薄弱环节，计划生育工作出现反弹，社会治安、安全生产压力较大，群众对食品药品安全还不够满意。政府职能转变不够到位，思维方式、行为习惯、工作方法还不能很好地适应新常态要求。有的干部工作作风不扎实，不敬业、不专业，甚至为官不为、为官乱为等。对上述问题，我们一定高度重视，在今后的工作中认真加以解决。

　　二、2015年目标任务

　　按照市委十二届十次全会的总体安排部署，今年我们要牢牢抓住第一要务不放松，以质量效益为中心，以深化改革为动力，坚持稳中求进、好中求快、又好又快，全面实施产业提升、基础突破、扶贫攻坚、和谐创建“四项工程”，突出培植“五大主导产业”，坚定不移抓好招商引资和项目建设，统筹城乡发展，重视生态文明，保障改善民生，为实现“科学赶超、后来居上”奠定坚实基础。

　　2015年我市经济社会发展的主要预期目标是：地区生产总值增长9.5%左右，公共财政预算收入增长10%左右，规模以上工业增加值增长13%左右，规模以上固定资产投资增长16%左右，社会消费品零售总额增长13%左右，进出口总额增长15%左右，城镇居民人均可支配收入、农民人均纯收入分别增长9%左右和10%左右，登记失业率控制在4%以内，人口自然增长率控制在9.2‰以内，全面完成省下达的节能减排任务。

　　围绕实现上述目标，重点抓好七个方面的工作：

　　（一）坚定不移膨胀经济总量。立足欠发达实际，千方百计培育新的经济增长点，全力以赴加快发展步伐。一是突出抓好项目建设。把项目建设作为扩大投资的第一抓手，将经济社会发展任务全部落实到具体项目上，以项目建设带动产业集聚、规模扩张。依托产业基础和资源条件，加强投资机会研究和项目策划论证，力争每个县区策划、储备过10亿元项目5个以上，全市储备生产性项目投资额800亿元以上，争取更多项目列入省重点、国家和省“十三五”规划。对已确定的200个市重点项目，拉出时间表，绘出路线图，全力以赴推动。二是着力推进精准招商。主动适应投资冲动弱化、环境约束加剧、优惠政策取消的新变化，把工作重点转移到搭建平台、优化服务、营造环境上来。重点抓好资源招商、以商招商、亲情招商、园区招商、盘活存量招商，努力实现由粗放招商向精准招商转变。力争各县区平均签约项目投资额200亿元以上，当年落地50%以上。三是激励全民创业。突出抓好大学毕业生、转业军人和返乡农民工自主创业，大力吸引在外能人和海归人才来菏创业，鼓励支持村干部、致富能人带头创业，积极引导广大农民本土创业，力争新增个体工商户5万户以上、私营企业6500家以上。引导县区和有条件的乡镇加快创建企业孵化中心、大学生创业园、创意农业园，着力抓好各类专业批发市场建设，努力为创业者搭建平台、拓展空间。认真落实省市支持民营经济发展的意见，加快推进投资项目库、劳动力资源信息库和培训基地建设，加大创业典型宣传力度，营造全民创业的浓厚氛围。四是积极发展外向型经济。支持企业参与境外资源开发，加强跨境电商和境外营销网络建设，强化品牌培育，优化出口结构，多元化开拓国际市场。加快推进直通式海关监管场站、公用型保税仓库建设，扩大能源资源性产品、先进技术设备和关键零部件进口，力争实现进出口总额40.5亿美元。积极组织参加香港山东周、台湾山东周等活动，着力引进一批行业领先、辐射带动力强的外资项目，力争实际利用外资2.3亿美元。五是精心组织经济运行。建立经济运行联席会议制度，加强对企业生产经营的实时监测分析，及时帮助企业解决运行中的困难和问题。积极推进政银企合作，突出抓好签约资金落实，强化金融对实体经济支持。督促每个县区重点培育3家以上上市后备企业，支持企业借壳上市或通过新三板转入主板上市，不断拓宽融资渠道。加大高端人才引进力度，加强企业家队伍培训，为企业发展提供有力的人才支撑。

　　（二）坚定不移推动转型发展。围绕推动产业转型升级，提高发展质量和效益，着力强化五大支撑。一是强化主导产业支撑。把工业主导产业作为转型发展的重中之重，围绕完善拉伸产业链，着力引进培育上下游企业及相关配套企业，推动产业从原料加工向终端产品演进、向价值链两端迈进。生物医药产业以发展高端高效成品药为主攻方向，力争尽快跨入“全国医药产业十强市”。能源化工产业以加快煤电一体化进程为重点，努力打造全国重要的综合性现代能源化工基地。机电设备制造产业以发展自主品牌和提高产业集中度为核心，尽快培育一批主营业务收入过百亿企业。农副产品加工产业重点培育壮大“领军型”龙头企业，推动粮油、果蔬、林业、畜禽、纺织等产业向精深加工发展。引导各县区立足自身优势，集中力量培育2-3个主导产业，努力形成特色发展、错位发展、竞相发展格局。二是强化重点企业支撑。把“四上”企业作为转型发展的基本力量，探索建立“小升规”服务补贴券制度，鼓励个体工商户加快成长为小企业，小企业尽快升级为规模企业。引导规模企业完善法人治理结构，建立现代企业制度，努力成为“规改股、股上市”的后续力量。全市重点抓好100家、县区各自抓好50家骨干企业培育，支持其上项目、搞技改、快转型，促使其裂变式增长，成为地方经济的重要支撑。从百强企业中筛选20家进行重点扶持，推动其强强联合、兼并重组、合资合作，尽快成长为具有核心竞争力的大型企业集团。三是强化产业园区支撑。把11个省级工业园区作为转型发展的重要载体，进一步完善发展规划，强化配套设施，增强其承载聚集能力。突出抓好园区集中供热中心和重点企业双回路电源建设，着力解决统一供水、供电、供热、供气和集中治污问题，配套建设教育、卫生、休闲等生活服务设施，不断满足企业多元化需求。全力推动市开发区、高新区高端发展、快速发展，大力推进创新企业培育、创新平台建设、科技成果转化和新兴产业聚集，促进开发区尽快创建为国家级开发区、高新区加快建设为“中国北方医药城”核心区，切实发挥好转型升级的龙头带动作用和示范引领作用。四是强化服务业支撑。把服务业作为转型发展的重要内容，坚定不移提质增量，力争服务业增加值占比提高1个百分点。重点发展商贸物流业。按照“一核两轴四区多中心”的总体布局，加快建设大型商贸物流园区、专业批发市场、骨干商贸物流企业和配套支撑项目，积极兴建总部物流园区，力争交易额过100亿元的商贸物流园区达到5个。大力发展电子商务业。尽快出台加快电子商务业发展的意见，建立常态化培训机制，着力培育一批示范基地、示范企业，打造一批淘宝村、淘宝镇。加快推进电商产业园建设，搞好网络优化、道路交通、加工仓储等配套服务，促进电子商务业集群集聚发展。繁荣发展文化旅游业。充分挖掘“一都四乡”、历史名人、非物质文化遗产等资源，强化文化与旅游的融合，重点打造一批文化旅游项目，加快发展乡村旅游，尽快把菏泽建成区域旅游节点城市和游客集散地。加快发展现代金融业，确保新增金融机构5家，新增非银行金融机构和相关服务机构20家以上。积极发展体育产业、健康产业、会展经济、服务外包、信息服务、文化创意等新兴业态，努力提升服务业发展层次和水平。五是强化科技创新支撑。把科技创新作为转型发展的关键抓手，确保科技研发投入占比达到1.2%，高新技术产业产值占比达到31.5%。推动市开发区、高新区加快建设国家级科技企业孵化器，8县区加快建设省级以上科技企业孵化器，做实鲁南药物研究院等科研院所，抓好中科院山东综合技术转化中心菏泽中心、山东省科学院菏泽分院、中国医药工业研究总院菏泽分院等平台建设，新增创新平台20家。鼓励支持企业深化产学研合作，积极承担国家和省各类科技计划，搞好科技成果和发明专利申报，努力突破关键核心技术，加快向高新技术企业迈进。

　　（三）坚定不移实现农业发展新突破。坚持农业基础地位不动摇，大力发展现代农业，促进农业由大到强转变。一是大力推进农产品基地建设。整合涉农资金和项目，配套完善桥涵闸、道路、机井等设施，加快推进高标准农田建设，力争粮食总产达到710万吨，实现“十二连增”。继续抓好巨鑫源6万亩芦笋、佳农果蔬15万亩大蒜、沙土食品5万亩花生和定陶、单县为主的15万亩山药等特色基地建设，不断扩大经济作物种植面积。积极推进农牧对接，大力发展“两羊一牛”等特色养殖业，力争标准化养殖示范场发展到100个以上，畜牧业总产值达到145亿元。二是大力推进产业化经营。积极培育农业新型经营主体，力争新发展家庭农场、股份合作农场200家，市级以上农民专业合作社示范社100家，农业产业化龙头企业20家。不断优化农机装备结构，进一步提高农作物耕种收综合机械化水平。三是大力推进“一村一品”发展。以5个国家级、5个省级“一村一品”示范村镇为引领，突出抓好30个“一村一品”专业乡镇、300个重点村试点，带动各乡镇立足地方特色、产业优势，重点建设3-5个“一村一品”示范基地，尽快形成竞相发展的良好局面。大力发展都市农业、城郊农业、观光农业、特色农业，通过多种形式实现农产品价值。加大新品种开发引进力度，搞好农产品商标注册和“三品一标”认证，加快进入国内外高端市场。四是大力推进牡丹产业化。认真落实牡丹产业发展扶持政策，深入推进“十百千万工程”，着力抓好千亩以上种植基地建设、龙头企业培育、新产品研发和国际品牌打造，加快推动出口牡丹加工区建设，全方位促进牡丹产业快速发展，力争产业产值增长30%以上。

　　（四）坚定不移提高城镇化水平。一是突出抓好重大基础设施建设。把基础设施建设提升到事关全局和战略的高度，紧抓国家和省扩大有效投资的机遇，大力实施“基础突破工程”，全力推进“十大基础设施项目”建设。菏泽机场取得国家民航总局选址批复，力争尽快具备开工条件；菏泽至曲阜城际铁路抓紧推动前期工作，争取早日开工建设；菏泽至兰考城际铁路项目，尽快协调解决在郑徐客专兰考南站预留接轨问题，加快推进预可研等前期工作；京九铁路客运专线商丘以北段，联合相关市积极争取早日纳入国家规划；菏徐铁路菏泽段尽快启动前期工作；德上高速巨野至单县段完成前期准备；德商高速鄄菏段力争基本具备通车条件；东新高速基本完成主桥工程；郓城新河航道完成省立项；新万福河航道完成前期准备，力争开工建设。二是着力加强市区建设与管理。加快理顺城建管理体制。在“统一编制城市总体规划、统一编制城建年度计划、统一组织实施土地出让”的前提下，研究制定市区城建管理事权划分实施方案，最大限度下放权力，加快构建“两级政府、三级管理、四级网络”的高效运作体系。尽快完善城市总体规划。坚持将定陶和“三区”一体化布局，加快编制城区路网、公共服务设施等专项规划和重点区域、重要地段的控规，搞好进城道路、城市外环、市区道路的布局规划，确保与菏泽机场、城际铁路、高速公路、万福河航道码头等重大基础设施有效衔接。大力推进城建重点工程。握紧拳头、集中财力，着力实施好“四个十工程”：即，围绕完善城市功能，提高承载能力，加快推进丹阳立交桥、火车站人防、垃圾发电厂、市区西部热源等“十大市政工程”；围绕畅通城市经脉，减少交通堵塞，打通八一路、平原路、点将台路、中山路、青年路等十条断头路；围绕提升城市形象，完善城市功能，综合改造提升西安路、刘民路、曹州路、太原路等十条道路；围绕城中村改造，提高城市化水平，加快推进中南花城片区、双河集片区、中北片区、绿地片区等“十大片区开发”。牢固树立质量意识、精品观念，注重历史传承、地方特色，努力打造留得住城市记忆、唤得起美丽乡愁、彰显花城特色的“人文城市”。进一步强化城市管理。继续推进数字化城市建设，切实加强市政设施的日常维护、环卫保洁和绿化养护，全面提升城市精细化管理水平。组织开展好店外经营、重车进城、车辆乱停乱放、私设广告等专项整治，推进市容市貌综合治理从主次干道向背街巷延伸。开展物业管理综合整治专项活动，切实提升小区物业服务水平。三是统筹推进县城和小城镇建设。深入推进郓城国家新型城镇化综合试点工作，引导各县加快县城基础设施和公共服务设施建设，着力增强产业集聚、人口承载和生活服务功能。年内8个县城全部实现管道燃气供应，新发展居民用户5万户。以全省开展“百镇建设示范行动”为契机，认真抓好省市扶持政策的落实，重点推进国家级综合改革试点镇、省级示范镇和市级重点镇建设，加快培育一批经济强镇、商贸重镇、旅游名镇。把农村新型社区逐步纳入城镇化管理，促进教育、卫生、商业连锁等公共服务向农村覆盖，推进道路、供水、环卫设施向农村延伸，逐步扩大建制镇驻地污水处理站覆盖面，所有乡镇建成垃圾中转站，实现城乡环卫一体化全覆盖。加快推进平原水库建设，力争纳入省规划的全部建成投用，为实现城乡供水一体化创造条件。积极采取PPP投融资模式，策划论证一批城镇供水供气、公共交通等基础设施和公共服务项目，吸引社会资本参与建设和运营。

　　（五）坚定不移深化重点领域改革。从群众关注的焦点着手，从制约菏泽发展的障碍改起，重点推进五个方面的改革。一是简政放权。加快制定行政权力清单、政府责任清单、市场准入负面清单，编制权力运行流程图，大力削减隐性附着在审批中的前置和服务事项，真正实现市场主体“法无禁止皆可为”。规范政务服务中心运行管理，深化“两集中、两到位”，杜绝多头办理、体外循环；降低收费标准，取消下放行政审批事项20%，再压缩审批时限30%，进一步提高审批效率和服务质量。二是事业单位改革。坚持事企分开、政事分开、管办分离，加快推进经营类事业单位转企改制，推进学校、医院等公益性事业单位去行政化，推进中介机构与挂靠行政事业单位彻底脱钩。健全政府购买服务目录体系，实现从花钱“养人”到“养事”转变。三是农村改革。健全完善市、县、乡三级农村土地流转服务平台，推动土地承包经营权有序流转。完成农村土地承包经营权确权登记颁证工作，探索土地承包经营权抵押、担保等办法，推动农业适度规模经营发展。稳步推进农村集体经济组织产权制度改革试点，积极探索建立综合性农村产权交易市场。四是金融体制改革。继续深化农信社银行化改革，确保鄄城、单县农商行挂牌运营。鼓励民间资本更大范围进入金融领域，规范引导民间融资机构和村镇银行、小额贷款、融资性担保等普惠金融组织快速发展。大力发展非银行类金融机构，支持发展金融租赁、消费公司、现代保险等新型金融业态。全面落实监管责任，防控系统性金融风险。五是财税体制改革。进一步深化预算管理改革，将政府收支全部纳入预算管理，市县两级政府和部门的“三公”经费预决算全部向社会公开。设立政府引导基金，实行专业化管理，吸引更多社会资本进入经济发展重点领域。探索实施以奖代补、民办公助等方式，鼓励引导社会力量兴办教育、医疗、养老等公益事业。改进政府采购方式，全面推进信息公开，提高采购绩效。全面推开“营改增”改革，稳妥实施煤炭资源税从价计征改革，确保地方财政收益。

　　（六）坚定不移加强生态文明建设。绿水青山就是金山银山。狠抓环境治理和生态保护，努力打造生态、美丽、宜居菏泽。一是加强科技节能减排。严格项目节能评估审查、新增能耗等量淘汰制度，从源头上控制能耗过快增长。深入开展能效对标活动，统筹抓好工业、建筑、交通等重点领域的节能，淘汰落后设备1000台套以上，实现节能30万吨标煤以上。大力推进节能、节水、节地、节材等新技术的推广应用，积极实施余热余压利用、绿色建筑等节能改造工程，以科技手段实行污染排放总量控制，确保“十二五”节能减排目标全面完成。二是加强资源集约节约利用。严厉查处违法违规用地，坚决守住耕地红线。严格用地审批和批后监管，加大闲置、低效利用土地清理整治力度，做好“腾笼换鸟”文章，提高土地利用效率，确保亿元GDP用地大幅降低。落实最严格的水资源管理制度，强化“三条红线”管理，加快推进节水型社会建设。大力实施清洁生产企业示范工程，着力推进循环经济示范园区建设，培育资源综合利用企业20家以上。三是加强生态环境保护。完善环境保护考核机制、生态补偿机制，健全网格化监管体系，深入开展环保专项行动，推动环保工作常态化、长效化。严把项目环评审批关，严格落实“三同时”制度，切实控制新增污染。健全大气治理联防联控机制，抓好重点领域、重点行业空气污染专项治理，确保“蓝天白云”天数明显增加。继续推行“河长制”，加强“治、用、保”水环境防控体系建设，切实解决污水直排问题。完善排污企业派员驻厂、环境违法案件有奖举报制度，强化日常监管和违法排污查处，加大刑责治污力度，确保大气和水环境质量持续改善。大力推进植树造林和城乡绿化，积极搞好农村坑塘治理，持续推进生态文明乡村建设，切实让人民群众感受到生态环境的明显变化。

　　（七）坚定不移保障改善民生。把人民群众对美好生活的向往作为政府工作的努力方向，千方百计保障和改善民生，努力让人民群众在发展中得到更多实惠。一是全力打好扶贫开发攻坚战。对扶贫对象实施动态管理，逐村逐户建档立卡、落实脱贫方案，实现扶贫开发差异化、精准化。加大财政专项资金投入，扎实做好驻村帮扶、教育扶贫等工作，深入开展党员干部联系困难群众、扶贫志愿者和慈善救助活动，鼓励引导企业、社会组织和个人通过各种方式参与扶贫开发。积极开展产业扶贫、专业扶贫行动，推进集中连片扶贫开发试点，结合镇村实际，大力发展种植、养殖、加工等农村特色产业，带动更多的农民在家门口就业创业、脱贫致富，年内实现40万人脱贫。二是大力实施“和谐创建工程”。创新社会治理方式，健全矛盾纠纷排查化解和突发事件应急救援机制，推动更多资源、服务、管理下沉到城乡社区。加快县级综合接访中心建设，完善领导干部公开接访制度，畅通群众诉求表达、利益协调、权益保障渠道。扎实推进“平安菏泽”建设，健全立体化治安防控体系，严厉打击各类违法犯罪活动。推行安全生产“网格化”管理和监管责任“实名制”，强化企业的主体责任，深入开展“打非治违”和安全隐患排查整治活动，加大对重点行业、重点领域的安全监管力度，严防各类事故发生。切实加强市场监管，严厉打击商业欺诈、制假售假和不公平竞争行为，努力营造公平、规范、有序的市场环境。加快构建食品药品监管和农产品质量安全追溯体系，着力治理“餐桌污染”，保障人民群众饮食用药安全。大力弘扬优秀传统文化，认真践行“崇文尚德、务实图强”的菏泽精神，让新风正气成为全市上下的共同价值追求。三是加快推进社会事业发展。优先发展教育事业。扎实推进校长职级制改革，加快推进“全面改薄”工程，积极推动义务教育均衡发展，努力让农村孩子享受到优质教育。加大教育投入，支持菏泽学院创建山东省应用型人才培养特色名校，争取齐鲁工业大学菏泽校区尽快开工建设，新建改建中小学校舍100万平方米以上、幼儿园200所、城区中小学10所以上，公开招录中小学、幼儿园教师2000名以上，普通高中、中职学校助学资助标准由每生1500元提高到2000元。大力发展职业教育，加强公共实训基地建设，加快培养实用性技术人才。抓好就业再就业工作，新增城镇就业9万人，农村劳动力转移就业18万人。大力发展养老事业。扎实推进菏泽医专养老基地建设，全面完成县级社会福利中心和中心敬老院建设，50%的城市社区建成城市老年人日间照料中心、30%的农村社区建成农村互助幸福院，每千名老人拥有养老床位达到30张。加大社保救助力度，城乡居民养老保险基础养老金、低保重度残疾人政府补助标准分别由每月每人75元、50元，提高到85元和80元；适当提高城乡低保、农村五保、大病救助标准；继续提高退休职工待遇；贫困寄宿生资助覆盖面扩大到15%。大力发展卫生计生事业。继续推进公立医院改革，巩固完善基本药物制度，健全县乡村三级公共卫生管理体系，不断提高医疗服务水平。继续免费为城乡居民提供11类43项基本公共卫生服务，人均基本公共卫生服务经费补助由35元提高到40元；继续提高城乡居民基本医疗保险财政补助标准，从每人每年320元提高到380元；严格落实计划生育基本国策，下大力控制出生人口性别比，促进人口均衡发展。加快发展文体事业。加强城乡公共文化基础设施建设，实施文化下乡活动和“乡村记忆工程”，积极发展“智慧社区”，新增乡镇综合性文化站示范点20家，新建农村文化广场1600个、覆盖率达到50%以上，新增村级文化团体1200个、覆盖率达到65%以上，送戏下乡1500场，送电影下乡6.6万场。完善全民健身服务体系，完成村级农民健身工程500个。四是集中力量办好10件民生实事。（1）解决127万农村人口的安全饮水问题。（2）开工建设市老年大学、老干部活动中心和全民健身中心。（3）新增新能源公交车100辆、公共自行车1000辆。（4）实现就餐千人以上学校食堂后厨亮化、快速检测。（5）救助贫困家庭大学新生5000名以上。（6）创建菏泽市创业实训孵化基地。（7）为全市50万名高血压患者、17万名糖尿病患者免费建立健康档案并提供保健医疗服务。（8）开工保障性安居工程1.8万套，完成农村危房改造5000户。（9）改造农村公路1000公里以上、大中型窄危桥30座以上。（10）投资2.5亿元实现24万亩高标准农田刷卡取水灌溉。

　　三、切实加强自身建设

　　按照“为民、务实、清廉”要求，切实强化法治政府和廉洁政府建设，努力打造人民满意的政府。

　　（一）坚持为民施政。以民之所望为施政所向，切实巩固群众路线教育实践活动成果，始终把群众观点植根于血脉里、体现到政策中、落实到行动上。坚持立身当伞、俯首为牛，深入开展“访民情、听民意、解民忧”活动，对群众多一份关心、多一份体贴，尽心竭力多办利民之事。完善社情民意收集机制，扎实办好市（县）长公开电话，用心解决好群众反映的切身利益问题。巩固优化营商环境成果，深入推进“两集中、两到位”，全面提升公共服务效能。推进县乡村三级便民服务中心建设，让群众方便办事成为新常态。各级政府都要始终与人民心心相印、与人民同甘共苦、与人民团结奋斗，努力向菏泽人民交一份合格的答卷。

　　（二）坚持依法行政。强化法治理念和法治思维，创新执法体制，完善执法程序，推进综合执法，严格执法责任，建立权责统一、权威高效的依法行政体制。主动接受市人大法律监督、工作监督和市政协民主监督，认真听取各民主党派、工商联、无党派人士和社会各界意见。把群众参与、专家论证、风险评估、合法性审查、集体决策作为重大决策的必经程序，以程序的缜密保证决策的科学。深化政务公开，实行决策、执行、管理、服务、结果全方位公开，坚决让权力在阳光下运行。始终坚持“法定职责必须为、法无授权不可为”，切实打通政策落实“最后一公里”，确保政令畅通、令行禁止。

　　（三）坚持务实勤政。牢固树立“功成不必在我”的理念，勇于担当、锲而不舍，多做打基础、利长远、惠民生的事情。强化责任意识，抓住机遇、用好政策，确保中央、省委、省政府和市委的部署落到实处、取得实效。进一步完善层层负责、科学合理、行之有效的工作机制，拉出时间表，绘出路线图，精准发力、持续发力，以钉钉子精神狠抓工作落实。大力弘扬雷厉风行、立说立行工作作风，说了算、定了干，干就干好，决不搞形式、放空炮、走过场。建立健全“横到边、纵到底”的责任体系和监督考核机制，坚决纠正不作为、乱作为，坚决克服懒政、怠政，坚决惩处失职、渎职，努力打造一支政治坚定、能力过硬、作风优良、勤勉尽责的人民公仆队伍。

　　（四）坚持廉洁从政。坚持党风廉政建设与经济社会发展同谋划、同部署、同考核，认真履行党风廉政建设责任制，筑牢拒腐防变的堤坝。认真遵守政治纪律和政治规矩，自觉践行“三严三实”要求，深入落实中央八项规定精神，横下一条心纠正“四风”，常抓抓出习惯、抓出长效。牢固树立过“紧日子”思想，精打细算，严管严控，确保“三公”经费只减不增。切实完善和规范行政监察、经济审计等制度，加强对项目招投标、工程建设、土地出让、政府采购等重点领域和环节的监管，有效制约权力运行。严格落实“两个责任”，坚持有腐必反、除恶务尽，以锲而不舍、驰而不息的决心，始终保持反腐败的高压态势，坚决查处各类违纪违法行为，决不让腐败分子有立足之地。

　　各位代表，菏泽正处在加快发展、跨越发展的关键时期，责任重大，使命光荣。让我们在中共菏泽市委的坚强领导下，紧紧依靠全市人民，凝心聚力，攻坚克难，开拓进取，扎实苦干，为实现“科学赶超、后来居上”的宏伟目标而努力奋斗！
